# Genetic diversity assessment of Tunisian *Mycobacterium bovis* population isolated from cattle

**DOI:** 10.1186/s12917-017-1314-y

**Published:** 2017-12-16

**Authors:** Saif Eddine Djemal, Mariam Siala, Salma Smaoui, Sana Kammoun, Chema Marouane, Javier Bezos, Feriele Messadi-Akrout, Beatriz Romero, Radhouane Gdoura

**Affiliations:** 1 Department of Life Sciences, Research Laboratory of Environmental Toxicology-Microbiology and Health (LR17ES06), Faculty of Sciences, University of Sfax-Tunisia, Sfax, Tunisia; 2 Department of Biology, Preparatory Institute for Engineering Studies, University of Sfax-Tunisia, Sfax, Tunisia; 3 Department of Microbiology, Regional Hygiene Care Mycobacteriology Laboratory, Hedi-Chaker University Hospital, Sfax, Tunisia; 4 Department of Biology, Faculty of Pharmacy, University of Monastir-Tunisia, Monastir, Tunisia; 5 Department of Microbiology, National Reference Laboratory of Mycobacteria, Research Unit (UR12SP18), A, Mami University Hospital of Pneumology, Ariana, Tunisia; 6 MAEVA SERVET SL. C/ de la Fragua 3, 28749, Alameda del Valle, Madrid, Spain; 7 Centro de Vigilancia Sanitaria Veterinaria (VISAVET), Universidad Complutense, Avda. Puerta de Hierro s/n, 28040 Madrid, Spain

**Keywords:** *Mycobacterium Bovis*, Genetic diversity, Spoligotyping, MIRU-VNTR typing

## Abstract

**Background:**

The genetic diversity of *M. bovis* in Tunisia is still underestimated despite the implementation of an eradication program. The lack of data about spatial distribution of the *M. bovis* population hinders the control of bovine tuberculosis (bTB) progress. This study represents the largest molecular analysis of *M. bovis* isolates in Tunisia. It is aimed to upgrade the understanding of bTB epidemiology and the geographical distribution of the infection. Tuberculosis research was performed in cattle (*n* = 149) with TB-compatible lesions collected over 5 months from a slaughterhouse located in Sfax, Tunisia.

**Results:**

Ninety-four animals were found to be infected by *M. bovis* and two others by *M. caprae*. Spoligotyping revealed twenty-five patterns, SB0120, SB0134, and SB0121 being the most prevalent profiles (36.4%, 11.4%, and 7.2%, respectively). Three new spoligotypes were detected: SB2345, SB2344 and SB2343. MIRU-VNTR analysis classified the isolates in seventy-three profiles and showed a large genotypic variety observed within the main spoligotype which was split into several MIRU-VNTR types: 29 in SB0120 (*h* = 0.983), 10 in SB0134 (*h* = 0.981) and 7 in SB0121 (*h* = 1). Genotyping revealed a common pattern in different geographic regions. It also showed that Sfax, located in southern-Tunisia, represents a high-risk area with an elevated genetic diversity.

**Conclusions:**

Spatial analysis may provide insights into disease transmission, which affects the effectiveness of eradication campaigns in cattle.

**Electronic supplementary material:**

The online version of this article (10.1186/s12917-017-1314-y) contains supplementary material, which is available to authorized users.

## Background

Bovine tuberculosis (bTB) is one of the seven endemic zoonoses neglected worldwide, especially in developing countries [1]. It is mainly caused by *M. bovis*, a member of the *Mycobacterium tuberculosis* complex (MTBC). *M. bovis* has a wide host range and can affect various target species, including domesticanimals, mainly cattle. *M. bovis* can be transmitted from animals to humans in close contact including aerosols, consuming unpasteurized milk or dairy products and less often by skin contact.

A national bTB control program has been implemented in Tunisia since 1984 based essentially on the intra-dermal tuberculin skin testing and routine slaughterhouse meat inspections of dairy cattle [2].

In fact, there are two livestock sectors characterized as follows:

- The organized intensive livestock belonging to the state and subjected to the bTB eradication program.
- The private sector is the so-called unorganized Tunisian livestock, which consists of 60 to 70% of the whole bovine livestock and extensive or semi-extensive cattle herds belonging to private owners [3]. Nevertheless, the bTB is still prevalent mainly in the private sector where disease control is based on a scarce veterinary activity limited to visual inspection of carcasses in slaughterhouses without routine intra-dermal tuberculin skin testing. From January to August 2013, 499 cows showed TB-compatible lesions during the meat inspection among 37,060 slaughtered animals [4]. Moreover, the majority of Tunisian cattle are not registered and cattle movement control schemes are unknown. Most of the bTB cases in Tunisia are discovered during meat inspection of cattle slaughtered at regular slaughterhouses when gross visible lesions typical of the disease are detected. However, in two recent Tunisian studies, *M. bovis* was isolated from milk and in 35% of the cattle exhibiting lesions [3, 5]. This suggests that bTB caused by *M. bovis* still persists in animals and thus could affect ecosystems and cause human extra-pulmonary tuberculosis (EPTB). In fact, in Tunisia, the prevalence of *M. bovis* as a causative agent of EPTB is high and divergent from studies in African countries [6]. Recently, Ghariani et al. and Siala et al. have shown that *M. bovis*, the agent of bovine TB, is a major cause of lymphadenitis TB with a frequency of 57% in north and 76% in south Tunisia, respectively [7, 8]. Consumption of not well-cooked meat and drinking raw milk from infected animals are common practices and could be the main causes of human EPTB [7, 8].

Spacer oligonucleotide typing (spoligotyping) and Variable Number of Tandem Repeat (VNTR) typing have been shown to be efficient tools to discriminate between *M. bovis* isolates [9, 10].

The ability to compare *M. bovis* isolates in the same country and among countries could help understand the distribution of *M. bovis* strains. In Tunisia, a large-scale study has not yet been possible since the two existing studies have analysed a low size of samples. In addition, based on the strong historical commercial links between Tunisia and France (the main cattle supplier of Tunisia), a thorough description of the genetic discrimination power of VNTR on French strains of the same spoligotyping group previously published by Hauer et al. [11] could clarify whether the French input to the Tunisian *M. bovis* population could exist?

Therefore, the main goal of the present work was to study the genetic diversity of a large *M. bovis* population using spoligotyping and VNTR typing and to identify a potential exchange of strains between Tunisia and France.

## Methods

### Samples

Lung, liver, and lymph nodes (retropharyngeal/mandibular, mediastinal/bronchial, portal and mesenteric) were collected from 149 dairy cattle (Frisonne-Holstein breed) with TB-compatible lesions during the veterinary inspection of carcasses. The samples were only taken from the private unorganized livestock sector slaughtered in the largest slaughterhouse in the south of Tunisia from November 2014 to April 2015. Animals belonged to ten geographic regions of Tunisia: Sfax (*n* = 63), Ariana (*n* = 1), Gabes (*n* = 3), Mahdia (*n* = 13), Sidi bouzid (*n* = 6), Kairouan (*n* = 1), Beja (*n* = 1), Jendouba (*n* = 1), Manouba (*n* = 4) and Monastir (*n* = 3). Cattle were 48 male less than 18 month old calves and 101 cull dairy cows aged more than 5 years. Data about municipality and city of origin were obtained from the Ministry of Agriculture according to a registration number given to each cattle.

### Bacterial isolation

Five to ten grams of lymph nodes or lung without fat were cut into small pieces (approx. 50–100 mg) and macerated in 20 mL sterile distilled water in a Stomacher (BagMixer) until an even suspension was obtained. Tissues from each animal were processed separately. An equal volume of 4% (*w*/*v*) NaOH was added to the aliquot of tissue suspension to give a final concentration of 2%. After the addition of NaOH, the suspension was vigorously shaken and then incubated at 37 °C for 30 min, after which time the suspension was neutralized with 10% H_2_SO_4_. The treated tissue suspensions were centrifuged at 3000 g for 20 min and then the supernatant was discarded. Culture media Colestos (Bio-Rad, France) and Lowenstein–Jensen (Bio-Rad, France) were inoculated with the sediment using a cotton swab. The applicator was recharged with sediment before each medium slope was inoculated. The inoculated medium was incubated at 37 °C for 12–15 weeks and examined weekly for the presence of colonies [4].

### Identification of MTBC by IS*6110*-PCR

A loopful of each isolate was harvested into 200 ul TE buffer (10 mM Tris-HCl, pH 8 ± 0; 1 mM EDTA) in screw-cap 1.5 ml microcentrifuge tubes and boiled for 20 min. After centrifugation in 9000 g for 20 min, the supernatant was transferred to fresh tubes. All DNA extracts were used as template for the identification of MTBC by amplifying the insertion sequence IS*6110*.

A conventional PCR for the detection of the MTBC was performed to test the DNA samples extracted as well as positive and negative controls, following a previously described protocol [12]. DNA was amplified using 1.25 units of Takara Ex Taq DNA polymerase (Takara *Ex taq*, Otsu, Shiga, Japan) and 1 μl of DNA extract. PCR was carried out in a Gene-Amp PCR System 9700 (Applied Biosystems, Foster City, CA, USA). PCR amplification products were visualized using ethidium bromide staining after agarose gel electrophoresis.

### Spoligotyping and MIRU-VNTR typing

The MTBC strains were spoligotyped as described by Kamerbeek et al. [13] using the spoligotyping membrane made by the VISAVET Health Surveillance Centre (Madrid, Spain). The spoligotyping patterns were assigned according to the Mbovis.org website [14].

MIRU-VNTR analysis was carried out as described by Roring et al. [15] amplifying the loci ETR-A and ETR-B [9], MIRU 4, MIRU 31, MIRU 26 [16, 17], and QUB 3232, QUB 11a, QUB 11b and QUB 26 [15, 18]. The amplification program consisted of 15 min at 95 °C, followed by 40 cycles of 30 s at 95 °C, 60 s at 60 °C, and 120 s at 72 °C. Amplicon sizes were estimated by electrophoresis on a 2.5% agarose gel at 45 V during 5 h, using 100-bp ladder (Biotools B&M Labs, Madrid, Spain) and the number of repeats was calculated. The VNTR profiles were named by a P followed by a correlated number (i.e. P1, P2, P3, etc.).

### Data analysis

A dendogram was built with the unweighted pair group method with arithmetic average (UPGMA) and NJ (Neighbour Joining) clustering methods using the MIRU-VNTR data using Tree graph 2 software. A group of two or more isolates sharing the same MIRU-VNTR pattern was defined as a cluster. The discriminative power of each typing method was calculated using the Hunter–Gaston diversity index (HGDI) [19] using the website application http://insilico.ehu.es/.

## Results

Among the 149 cattle samples with suspicious TB lesions collected between November 2014 and April 2015, live mycobacteria were detected by culture in 99 animals (66.4%), 96 isolates belonged to *M. tuberculosis* complex and three to atypical mycobacteria. No coinfection with other strains was found in any sample.

**Fig. 1:**
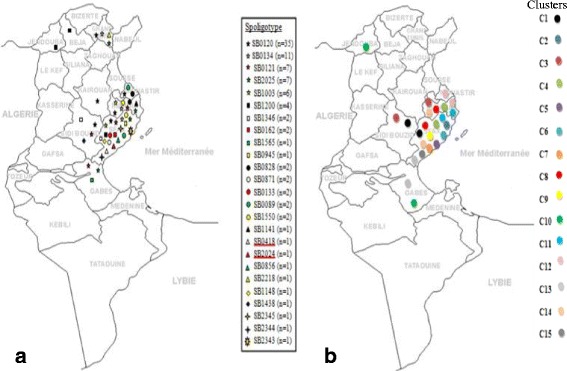
Geographical distribution of clusters and spoligotypes. **a** Distribution of twenty five spoligotypes distributed on ten Tunisian regions. The two *M.caprae* spoligotypes are underlined with red color. **b** Distribution of fifteen clusters identified using the UPGMA algorithm

**Fig. 2:**
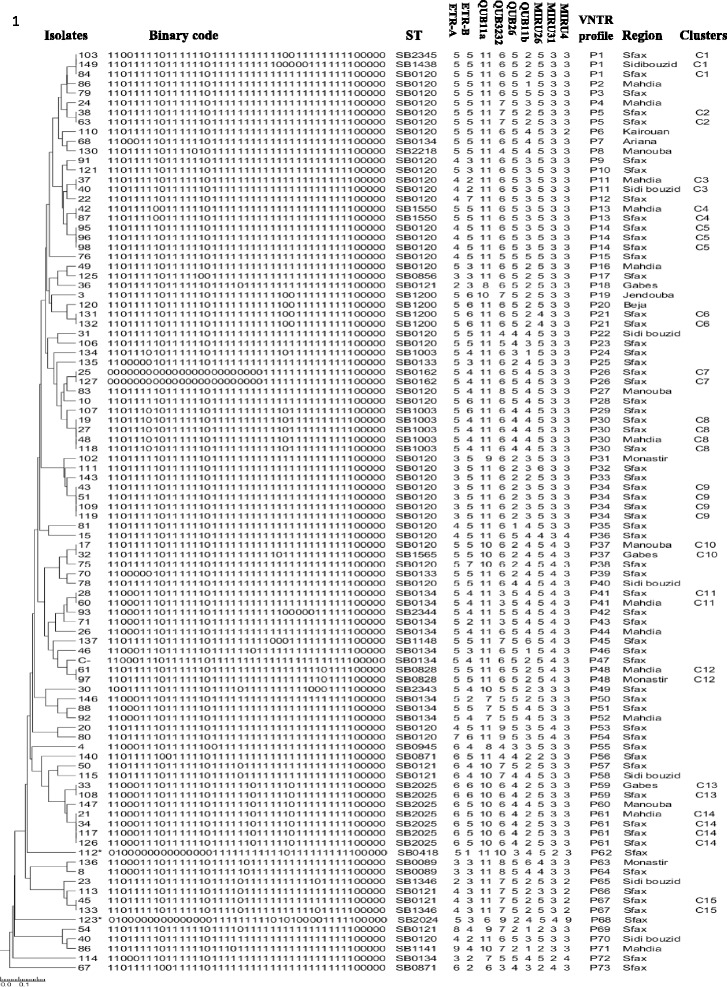
Dendogram of cluster analysis. Dendrogram generated with the UPGMA (unweighted pair group method with arithmetic average) and Neighbour Joining (NJ) clustering methods using the MIRU-VNTR data using Tree graph 2 software showing the genetic relationships of the 96 *Mycobacterium bovis* isolates. A group of two or more isolates sharing the same MIRU-VNTR pattern is defined as a cluster. ST: Spoligotype as defined in the *M. bovis* Spoligotype Database website (http://www.mbovis.org). * Isolate corresponding to *M. caprae*

Three profiles were typical of *M. bovis*, and two were classified as *M. caprae* (SB2024 and SB0418 patterns) (Figs. 1 and 2). The discriminatory power of spoligotyping was *h* = 0.842.

The most predominant spoligotypes were the SB0120 (*n* = 35, 36.4%), SB0134 (*n* = 11, 11.4%), and SB0121 (*n* = 7, 7.2%). Three new spoligotyping profiles were described (SB2345, SB2344, and SB2343). The geographic distribution of the different spoligotypes is shown in Fig. 1. The region of Sfax showed a very high level of genetic diversity with 20 out of the 25 detected spoligotypes.

The frequency of the three most detected spoligotypes in our strains (SB0120, SB0121, SB0134) was quite similar to those presented in Tunisia by Lamine-Khemiri et al. [3] and are also the three most frequent types observed in France by Hauer et al. [11] (Additional file 1).

All the 96 MTBC isolates were genotyped using nine MIRU-VNTR loci. Seventy-three different VNTR profiles were found (Fig. 2) and two of them were associated to the *M. caprae* isolates (P62 and P68). The discriminatory power for the MIRU-VNTR technique was high (*h* = 0.992). The discriminatory power of the nine individual loci ranged from 0.158 to 0.727. The loci with higher diversity were QUB-11b, ETR-B, ETR-A, QUB-3232, QUB26 and QUB-11a with a discriminatory power *h* corresponding to 0.727, 0.724, 0.650, 0.583, 0.577, and 0.445, respectively. The MIRU 31, MIRU26 and MIRU 4 loci displayed a very little allelic diversity (0.370, 0.249, and 0.158, respectively).

Our results showed a large genotypic variety observed within the main spoligotypes which was split into several MIRU-VNTR types as previously shown in Tunisia and in France [3, 11]. MIRU-VNTR typing results for 6 common loci (ETR A, ETR B, MIRU4 (ETRD), QUB11a, QUB11b and QUB3232) were available for strains from Tunisia and France showing shared genotypes (Additional file 1).

MIRU-VNTR analysis showed a large genotypic variety observed within the main spoligotype that was split into several MIRU-VNTR types: 29 in SB0120 (*h* = 0.983), 10 in SB0134 (*h* = 0.981) and 7 in SB0121 (*h* = 1). Genotyping revealed a pattern common for different geographic regions (P11, P13, P30, P41, P48, P59 and P61) (Fig. 1, Fig. 2). Our results showed that Sfax, located in southern Tunisia, represents a high-risk area with a high level of genetic diversity with 20 and 63 out of the 25 and the 73 detected spoligotypes and genotypes, respectively.

Fifteen clusters including 36 isolates (37.5%) were identified using the UPGMA algorithm and the largest three clusters (C1, C10 and C15) had four isolates; while most clusters had only two (Fig. 2). The most common VNTR profiles P30, P34 and P61 were found in four isolates each. Moreover, most clusters showed the same spoligotyping pattern for all isolates. Isolates with the spoligotyping patterns SB1438, SB2345, and SB0120 presented a similar MIRU-VNTR profile (P1: 5.5.11.6.5.2.5.3.3). It also occurred in the case of isolates with spoligotypes SB0120 and SB1565 where MIRU-VNTR profiles were P37: 5.5.10.6.2.4.5.4.3, and in isolates with spoligotypes SB1346 and SB0121 (MIRU-VNTR profile P67: 4.3.11.7.5.2.5.3.2) (Fig. 2). When both typing techniques were combined, the level of discrimination was 0.993 and 78 different spoligo–MIRU–VNTR types were observed.

For SB0120, the main MIRU-VNTR pattern (MV16), 5.5.3.11.4.6 identified in our study was detected in the Central East of France in the Côte d’Or region. Additionally, pattern MV6 (5.5.3.11.4.4) was identified for strains with SB0120 from France and Tunisia. Interestingly, four genotypes (MV21, MV10, MV53 and MV63) detected in our strains with an SB0120 spoligotype pattern from Sfax were previously described by Lamine-Khmiri et al. in the same region [3] but not detected in strains from France [11] (Table 1, Additional file 1). Moreover, MIRU-VNTR genotype MV53 (6.5.3.10.2.6) was detected according to our study in strains corresponding to the spoligotype SB2025; which highlights matching results only with the study conducted by Lamine-Khmiri et al. [3] (Table 1, Additional file 1). Interestingly, the pattern MV36 (5.4.3.11.4.6) was obtained in strains with spoligotype pattern SB0134 from Tunisia and France.

**Table 1:** Shared MIRU-VNTR genotypes of strains with spoligotype pattern SB0120 from Tunisia and France

	Tunisia		France
VNTR type	This study	Lamine-Khemiri et al. [3]	Hauer et al. [11]
MV6	5.5.3.11.4.4^£^	5.5.3.11.4.4^£^	5.5.3.11.4.4^£^
MV16	5.5.3.11.4.6^€^		5.5.3.11.4.6^€^
MV21	5.5.3.11.2.6^¥^	5.5.3.11.2.6^¥^	
MV10	3.5.3.11.3.6^¥^	3.5.3.11.3.6^¥^	
MV53	6.5.3.10.2.6^¥^	6.5.3.10.2.6^¥^	
MV63	5.6.3.11.2.6^¥^	5.6.3.11.2.6^¥^	

Results from Tunisia and France have previously been published. [3, 11] Additional file 1 shows more details concerning the comparison of our results to those of two previous studies. £Matching patterns of strains from the two Tunisian studies and France. € Matching patterns of strains from our study and France. ¥ Matching patterns of strains from our study and Lamine-Khemiri et al. MV53 genotype was also detected in strains with SB2025 genotype in our study and in Tunisia previously published by Lamine-Khemiri et al

## Discussion

Our study represented a large analysis including 96 characterized *M. bovis* out of 149 slaughtered cattle from ten Tunisian regions showing TB compatible lesions in the lymph nodes after routine inspection. However, two Tunisian studies have previously reported data about bTB in Tunisia analyzing only 35 and 6 *M. bovis* isolates out of 100 cattle and 306 milk samples, respectively [3, 5]. The allelic diversity of spoligotype patterns among the *M. bovis* strains isolated in our study (*h* = 0.842) were relatively more than the ones previously found by Lamine-Khemiri et al. and Kahla et al. (0.733 and 0.753, respectively) in Tunisia [3, 5]. This fact could be mainly due to the differences in the number of isolates.

Of the 25 detected *M. bovis* spoligotype patterns, 8 patterns, accounting for 75% of all the isolated strains, have previously been detected in strains isolated from Tunisia [3]. The frequency of three common spoligotypes most detected in our strains (SB0120, SB0121, SB0134) are quite similar to those detected in Tunisia by Lamine-Khemiri et al. [3], in Algeria by Sahraoui et al. [20] are also in France by Hauer et al. [11]. They were also found in other European countries as Italy, Spain and Portugal [10, 21, 22]. Our present results corroborate previous studies showing a more frequent occurrence of *M. bovis* strains of spoligotype pattern SB0120 causing bTB in Tunisia (36–37%), in Algeria (39%) [20] and in France since 1978 accounting for 26% as mentioned by Hauer et al. [11]. Interestingly, the spoligotype SB0134 was identified as the second predominant type in Tunisia, the second most frequently isolated type in France [11] and was highly prevalent in cattle from neighbouring Algeria [20]. SB0121,the third most abundant spoligotype in our study (7.2%), is also present in France (6%) [11], and more frequently found in Algeria (21%) [20].

The comparative study of the Tunisian and French spoligotypes and MIRU-VNTR profiles could further support the French input to the Tunisian *M. bovis* population considering the striking similarity. There have been very strong historical commercial links between Tunisia and France starting during the French colonial period. The importation of French cattle could explain the majority of our genotypic results and the similarity with the *M. bovis* French population genotypes is concerned. The Food and Agriculture Organization in Tunisia, states documentation concerning the imported breeds in Tunisia originated from North America and Europe only from 1986 to 1989 [23]. Therefore the increased effort to control bTB in Europe or North America had already allowed almost eradication of the disease [24]. Thus, introduction of *M. bovis* from Europe into African countries has previously been suggested [10, 25]. In addition, Sahraoui et al. [20] mentioned that some strains of *M. bovis* found in Algeria may have been independently introduced from France (or more generally continental Europe). In fact, in Tunisia, the introduction of cattle from neighboring countries such Algeria and Lybia via non controlled routes is continuing up to date; which is considered one of the main sources of the maintenance of the infection in Tunisian cattle due to the absence of control and eradication campaigns. In addition, there are exclusive Tunisian spoligotypes clearly derived from strains SB0120, SB0134 or SB0121; some of which have already been described [11, 20], suggesting that the imported strains mutated in Tunisia. On the other hand, *M. bovis* mutates rather slowly; it is then highly possible that these mutated strains have long existed in the territory and that possibly spillback to the breed animals was introduced in the country.

The origin farms of the animals could not be traced since samples were taken only from cattle belonged to the private unorganized livestock characterized by a multiple selling of the animals prior to slaughter. However, the majority of animals could be dairy cattle; thus it is very improbable that animals had come from the same herds. Thus, our study demonstrates the importance of the elaboration of a tracing system which could be a useful tool for bTB monitoring.

Our findings emphasize previous claims that cattle could be infected by *M. caprae* spoligotypes (SB0418, SB2024) [3, 6]. Interestingly, the SB2024 profile has recently been described in Tunisia [3] whereas the spoligotype SB0418 has been mentioned in Central and Eastern European countries [10, 26, 27] as the most frequent spoligotype found in human and animal isolates [3, 27]. The current study raises the issue of a cross contamination in and between cattle and goats during their breeding or importation. In Tunisia, the herd of goats was estimated at 1.5 million head. In the North, goats are reared in extensive mixed farming systems with sheep and cows. Meanwhile, almost 60% of the goats are located in the Center and South of Tunisia, reared in semi-intensive oasis systems, in small herds [28]. However, to our knowledge, no data is available about the importation of small ruminants in the country although the introduction of *M. caprae* infected cattle and/or goats from other countries could be possible.

The spatial distribution of the *M. bovis* strains reveals the existence of isolates with shared spoligotypes and MIRU-VNTR profiles between different geographic Tunisian regions: Sfax and Gabes (P59), Sfax and Mahdia (P13, P30, P4 and P61), Mahdia and Monastir (P48), and Mahdia and Sidi Bouzid (P11), which revealed the movement of animals between them. This exchange event is probably related to economic facts like the trade of infected animals and the geographical distribution in the area. In fact, Sfax, Gabes, Mahdia, Sidi bouzid, and Monastir regions are contiguous states which may facilitate the movement of cattle among them.

Isolates corresponding to the cluster C1 (SB0120, SB1438 and SB2345 spoligotypes), showed the same MIRU-VNTR allelic profile, and SB1438 and SB2345 could be probably derived from a SB0120 isolate. In a similar way, isolates corresponding to clusters C10 and C15 have identical MIRU-VNTR allelic profiles, and the loss of a single spacer could be compatible with a one-step evolution (SB0120 → SB1565; SB0121 → SB1346, respectively) asmentioned by Romero et al. [29]. It has also been hypothesized that the appearance of new strains diverged from pre-existing clones which have lost spacers [29–32]. However, the molecular techniques used in our study cannot determine the oldest strains. Thus, further analysis by whole genome sequencing would be interesting for this purpose.

## Conclusions

The molecular data obtained until now would greatly support and provide hints to improve the national eradication campaign since it helps to determine the origin of outbreaks and better understand the link in-between btB transmission. Further laboratory testing, periodic sampling from different slaughterhouses and additional herd epidemiological studies should be conducted to determine the exact bTB prevalence and to further understand the mode of transmission.

## Additional file

Additional file 1: Molecular typing data comparison of *M. bovis* isolates in different regions in Tunisia and in France. The data provided shows common spoligotypes prevalence and MIRU-VNTR typing results for 6 common loci (ETR A, ETR B, MIRU4 (ETRD) for strains isolated from Tunisian and French regions. (PDF 114 kb)
