# Supplementary material for: Genetic diversity assessment of Tunisian Mycobacterium bovis population isolated from cattle
Source: BMC Vet Res. 2017 Dec 16;13:393. doi: 10.1186/s12917-017-1314-y (PMC5732386; doi:10.1186/s12917-017-1314-y)
Supplement: Additional file 1: — Molecular typing data comparison of M. bovis isolates in different regions in Tunisia and in France. The data provided shows common spoligotypes prevalence and MIRU-VNTR typing results for 6 common loci (ETR A, ETR B, MIRU4 (ETRD) for strains isolated from Tunisian and French regions. (PDF 114 kb) [file 12917_2017_1314_MOESM1_ESM.pdf]

Additional file 1: Molecular typing data comparison of *M. bovis* isolates in different regions in Tunisia and in French

Study	Source, (Nb)	Nb isolates *	Region/ Nb	Total of genotypes	Common VNTR loci/different Genotypes						VNTR types	HGDI	Total of cluster	Total of spoligotypes	Common frequent spoligotypes							
					ETR-A	ETR-B	MIRU4	QUB11a	QUB11b	QUB3232					SB 0120 Nb (%) Region (Nb) [Nb of genotype]	SB0134 Nb (%) Region (Nb) [Nb of genotype]	SB 0121 Nb (%) Region (Nb) [Nb of genotype]	SB 2025 Nb (%) Region (Nb) [Nb of genotype]	SB 0418 Nb (%) Region (Nb) [Nb of genotype]	SB 2024 Nb (%) Region (Nb) [Nb of genotype]	SB1003 Nb (%) Region (Nb) [Nb of genotype]	SB1200 Nb (%) Region (Nb) [Nb of genotype]
This study	Cattle, (149)	96	k, B, Je, Ma, Mo, A Sf, G, M, SB :10	73	5	6	3	11	4	6	MV1	0,993	15	25	35<br>(36.4%)<br>Sf(24)<br>M(4)<br>Ma(2)<br>Sb(3)<br>Mo(1)<br>k(1)<br>[29]	11<br>(11.4%)<br>Sf(7)<br>M(3)<br>A(1)<br>[10]	7<br>(7.2%)<br>Sf(4)<br>G(1)<br>M(1)<br>SB(1)<br>[7]	7<br>(7.2%)<br>Sf(4)<br>G(1)<br>Ma(1)<br>M(1)<br>[3]	1<br>(1.04%)<br>Sf(1)<br>[1]	1<br>(1.04%)<br>Sf(1)<br>[1]	6<br>(6.25%)<br>Sf(5)<br>M(1)<br>[3]	4<br>(4.1%)<br>Sf(2)<br>Je(1)<br>B(1)<br>[3]
					5	5	3	10	4	6	MV2											
					4	5	3	11	3	9	MV3											
					4	7	3	11	3	6	MV4											
					5	5	3	11	3	7	MV5											
					5	5	3	11	4	4	MV6											
					4	2	3	11	3	6	MV7											
					5	5	3	11	2	7	MV8											
					4	2	3	11	3	6	MV9											
					3	5	3	11	3	6	MV10											
					5	3	3	11	2	6	MV11											
					2	3	2	11	2	7	MV12											
					5	5	3	11	2	7	MV13											
					5	7	3	10	4	6	MV14											
					4	5	3	11	5	5	MV15											
					5	5	3	11	4	6	MV16											
					5	5	3	11	5	6	MV17											
					7	6	3	11	3	9	MV18											
					4	5	3	11	4	6	MV19											
					5	4	3	11	4	8	MV20											
					5	5	3	11	2	6	MV21											
					5	5	3	11	1	6	MV22											
					4	3	3	11	3	6	MV23											
					4	5	3	11	3	6	MV24											
					4	5	3	11	3	6	MV25											
					4	5	3	11	3	6	MV26											
					3	5	3	9	3	6	MV27											
					5	5	3	11	3	5	MV28											
					4	3	2	11	2	7	MV29											
					5	5	2	11	4	6	MV30											
					5	4	3	11	4	6	MV31											
					5	5	3	10	4	6	MV32											
					4	5	4	11	4	6	MV33											
					5	3	3	11	3	6	MV34											
					3	5	3	11	2	6	MV35											
					5	4	3	11	4	6	MV36											
					5	4	3	11	4	3	MV37											
					5	3	3	11	1	6	MV38											
					5	4	3	11	4	3	MV39											
					6	4	3	8	3	4	MV40											

Study	Source, (Nb)	Nb of isolates *	Region/ Nb	Total of genotypes	Common VNTR loci/different Genotypes							HGDI	Total of cluster	Total of spoligotypes	Common frequent spoligotypes							
					ETR-A	ETR-B	MIRU4	QUB11a	QUB11b	QUB3232					SB 0120 Nb (%) Region (Nb) [Nb of genotype]	SB0134 Nb (%) Region (Nb) [Nb of genotype]	SB 0121 Nb (%) Region (Nb) [Nb of genotype]	SB 2025 Nb (%) Region (Nb) [Nb of genotype]	SB 0418 Nb (%) Region (Nb) [Nb of genotype]	SB 2024 Nb (%) Region (Nb) [Nb of genotype]	SB1003 Nb (%) Region (Nb) [Nb of genotype]	SB1200 Nb (%) Region (Nb) [Nb of genotype]
					5	2	3	11	4	3	MV41											
					5	5	3	7	4	5	MV42											
					5	4	3	7	4	5	MV43											
					3	2	4	7	4	5	MV44											
					5	2	3	7	2	5	MV45											
					2	3	3	8	2	6	MV46											
					4	3	2	11	2	7	MV47											
					6	4	3	10	2	7	MV48											
					8	4	3	9	1	7	MV49											
					9	4	3	9	1	7	MV50											
					4	3	2	11	2	7	MV51											
					6	4	3	10	4	7	MV52											
					6	5	3	10	2	6	MV53											
					6	6	3	10	2	6	MV54											
					5	5	3	11	2	6	MV55											
					6	6	3	10	2	6	MV56											
					6	2	3	6	3	3	MV57											
					6	5	3	11	2	4	MV58											
					6	5	3	10	4	6	MV59											
					5	1	3	11	4	10	MV60											
					5	3	9	6	4	9	MV61											
					5	6	3	10	2	7	MV62											
					5	6	3	11	2	6	MV63											
					9	4	3	10	1	7	MV64											
					5	1	3	11	4	10	MV67											
					5	3	9	6	4	9	MV68											
					5	5	3	11	6	7	MV69											
					5	6	3	11	4	6	MV70											
					5	4	3	11	1	6	MV71											
					2	3	2	10	2	7	MV72											
					5	4	3	10	2	5	MV73											

Study	Source, (Nb)	Nb of isolates *	Region/Nb	Total of genotypes	Common VNTR loci/different Genotypes							HGDI	Total of cluster	Total of spoligotypes	Common frequent spoligotypes							
					ETR-A	ETR-B	MIRU4	QUB11a	QUB11b	QUB3232					SB 0120 Nb (%) Region (Nb) [Nb of genotype]	SB0134 Nb (%) Region (Nb) [Nb of genotype]	SB 0121 Nb (%) Region (Nb) [Nb of genotype]	SB 2025 Nb (%) Region (Nb) [Nb of genotype]	SB 0418 Nb (%) Region (Nb) [Nb of genotype]	SB 2024 Nb (%) Region (Nb) [Nb of genotype]	SB1003 Nb (%) Region (Nb) [Nb of genotype]	SB1200 Nb (%) Region (Nb) [Nb of genotype]
L-K et aL [3]	Cattle, (100)	35	Sf, G, B, SB, N :5	19	4	3	3	11	3	5		0.969	6	9	16 (37.1%)<br>Sf(11) B(2) SB(2) G(1)<br>[10]	7 (20%)<br>Sf(7)<br>[2]	1 (2.8%)<br>Sf(1)<br>[1]	3 (8.57%)<br>Sf(3)<br>[1]	2 (5.71%)<br>Sf(2)<br>[1]	1 (2.8%)<br>N(1)<br>[1]	2 (5.71%)<br>Sf(2)<br>[1]	1 (2.8%)<br>Sf(1)<br>[1]
					4	5	3	11	3	7												
					4	2	3	9	3	5												
					4	5	2	11	4	5												
					4	5	2	11	5	5												
					4	5	3	11	5	5												
					5	5	3	11	2	6	MV21											
					3	5	3	11	3	6	MV10											
					6	5	3	10	2	6	MV53											
					5	5	3	11	4	4	MV6											
					5	4	3	11	4	6	MV36											
					5	2	3	6	4	5												
					6	4	3	11	2	7												
					5	6	3	11	2	6	MV63											
					5	1	3	11	4	11												
					5	3	9	6	4	8												
					9	2	3	7	9	1												
BK et al [5]	Milk, 306	6	Mo, Tu, S : 3	4	5	4	4	12	4	--		--	1	3	0	0	0	0	0	0	2 (33.3%)<br>[1]	1 (16.6%)<br>[1]
					5	8	4	12	3	--												
					5	4	4	10	3	--												
					5	4	4	12	3	--												
Hauer et al [11]	Cattle (88%), wild animals (9%), farm animals (3%)	4,020	Ardennes ; Ariège ; Côte d'Or ; Corsica Island ; Dordogne & Charente ; Dordogne ; Normandy ; Atlantic ; Pyrenees ; Landes ; Lot et Garonne	706	5	3	6	11	4	8		---	--	176	778 (26%)<br>[154]	270 (11%)<br>[53]	154 (6%)<br>[50]	--	--	--	--	--
					5	5	3	11	4	6	MV16											
					4	5	3	11	4	7												
					5	5	3	11	4	4	MV6											
					5	3	3	9	4	6												
					6	5	3	6	4	6												
					7	4	3	10	4	10												
					5	4	3	11	4	6	MV36											
					6	4	3	6	4	6												
					6	4	3	11	2	7												
					5	2	3	8	2	6												

N° : Number ; : Only M.bovis; Sf : Sfax ; G : gabes ; M : Mahdia, SB : Sidi bouzid, K : kairaouen, B : Beja, Je : Jendouba, Ma : Manouba, Mo : Monastir, T : Tunis, N : Nabeul, A : Ariana. £Common region between the three Tunisian studies are in fold. MV: MIRU-VNTR.*
